# Dairy cow health across an intensification gradient in Europe: an integrated multi-indicator assessment

**DOI:** 10.3389/fvets.2026.1855637

**Published:** 2026-07-20

**Authors:** Maeva Crémilleux, Juliette Cuenot, Thierry Buronfosse, Laura Vogel, Alexander Starke, Audrey Michaud

**Affiliations:** 1 Agroécologie et Environnement, ISARA, Lyon, France; 2 VetAgro Sup, Lyon, France; 3 Laboratoire de Pathologie Clinique, VetAgro Sup, Lyon, France; 4 Klinik für Klauentiere, Veterinärmedizinische Fakultät der Universität Leipzig, Leipzig, Germany; 5 Université Clermont Auvergne, INRAE, VetAgro Sup, UMR Herbivores, Saint-Genès-Champanelle, France

**Keywords:** animal health, animal observation, dairy farming systems, grazing systems, integrated welfare, metabolic indicators

## Abstract

Dairy farming systems in Europe range from low-input grazing systems to highly specialized indoor production systems, raising important questions about the relationship between production strategies, animal health and the sustainability of dairy production. While previous studies have generally focused on isolated health or production traits, this study provides an integrated multi-indicator assessment of dairy cow health across three farming systems representing a gradient of production intensification: low-input grazing systems, moderate-input grazing systems (GraCow), and fully indoor systems. Data were collected from dairy farms in France and Germany between 2018 and 2022 using herd-level performance indicators, clinical observations of animals, milk quality indicators and blood metabolites biomarkers. Low input farms were characterized by smaller herd sizes (40.7 Livestock Units vs. 197.4 and 608.8 LU; *p* < 0.001), lower milk yield per cow (4,573 vs. 9,531 and 9,965 kg/cow/year; *p* < 0.001), and greater breed diversity than GraCow and Indoor systems, which were composed almost exclusively of Holstein cows. Compared with the two other systems, cows from low input farms showed shorter calving intervals (391 vs. 416 and 413 days), lower lameness scores (1.04 vs. 1.48 and 1.86; *p* < 0.01), higher body condition scores during lactation (3.22 vs. 2.37 and 2.58; *p* < 0.01), and lower culling rates. Metabolic differences were also observed, including lower *β*-hydroxybutyrate concentrations in low-input systems (*p* = 0.02), although most biomarkers remained within physiological reference ranges. Because breed composition differed between systems, the potential contribution of genetic background to health resilience was considered when interpreting the results. Overall, this study highlights the value of integrating complementary indicators to better characterize dairy cow health and supports the potential role of lower-input grazing systems in sustainable dairy production.

## 1 Introduction

Today, dairy farming faces several interrelated challenges, including the need to ensure economic viability, animal welfare, environmental sustainability, and public health (1–3). These challenges are closely linked to major environmental concerns such as greenhouse gas emissions, biodiversity loss, and the depletion of natural resources (4–6). At the same time, the global emergence and spread of infectious diseases, particularly in regions previously unaffected (e.g., the spread of bovine viral diarrhea in Europe), has further highlighted the central importance of animal health in livestock production systems.

In Europe, dairy production systems are highly diverse. Low-input systems based primarily on grazing coexist with mixed systems combining grass feeding and grazing, as well as highly specialized indoor systems relying largely on maize silage. This diversity reflects different responses to food production and territorial challenges, including food self-sufficiency, export-oriented production, and the maintenance of agricultural activity in rural areas. These systems are embedded within a wide range of agricultural models, from agroecological and organic systems to more intensive production systems (7). Dairy farming systems also differ considerably in terms of herd management practices. Differences include the breeds used, ranging from high-producing breeds such as Holstein to local breeds with lower production potential, as well as herd size. Herd sizes vary widely across Europe, from medium-sized herds of around 60–80 cows in countries such as France and Ireland to several hundred animals in countries such as Denmark, and in some cases more than one thousand cows in large German farms. Milk yields also vary substantially, ranging from approximately 3,200 liters per cow in Romania to around 9,500 liters per cow in Denmark, with additional variability observed within countries (8). France and Germany illustrate part of this European diversity, as they encompass contrasting dairy production systems ranging from grass-based, agroecological systems to more intensive indoor production systems. Moreover, Germany and France are the two leading milk-producing countries in Europe and, together with Poland, the Netherlands, Italy and Ireland, account for more than 70% of total European Union milk with production (9).

These structural and management differences result in a wide range of production intensification. Agricultural intensification is generally defined as an increase in agricultural production per unit of input, such as labor, land, time, fertilizer, feed, or capital (10, 11). The effects of dairy intensification and increased milk yield on bovine health have been extensively studied. High-producing cows are often associated with impaired immune function, which can increase the risk of production-related diseases such as mastitis or ketosis and may be accompanied by reduced reproductive performance (12–15). Several studies have also reported a higher prevalence of lameness among cows with higher milk yields compared lower-producing animals (16, 17). However, these associations likely reflect a combination of physiological and management-related factors rather than a direct causal effect of milk production itself. Fleischer et al. (18) identified associations between milk yield in the preceding lactation and the incidence of retained placenta, mastitis, and milk fever, while relationships between milk yield in the current lactation and conditions such as ovarian cysts, claw disorders, and milk fever have also been reported. Comparative research examining conventional and organic dairy systems has provided additional insights into the relationship between production intensity and herd health. A recent review by Bareille et al. (19) reported that the overall frequency of health disorders tends to be slightly lower in organic systems compared with conventional systems. Differences in mastitis prevalence were generally small, whereas metabolic disorders such as ketosis were often substantially reduced, with decreases of 50–75% reported in some analyses. Moreover, increasing the proportion of grazed herbage in dairy diets, which typically reflects lower production intensity, has been associated with reduced veterinary interventions, lower use of curative pharmaceuticals, decreased incidence of severe hoof lesions, improved longevity, and reduced perinatal mortality (20). Similarly, Rittweg et al. (21) reported that organic farming systems, particularly those providing access to pasture, are associated with a lower prevalence of lameness. Although considerable variability exists among organic herds across Europe (22), the available evidence suggests that herd health status is influenced by the level of production intensification. Grass-based and agroecological systems often exhibit more favorable health outcomes than highly intensive systems, highlighting the potential of lower-input strategies to support improved dairy cow health.

Assessing animal health requires a multidimensional approach. Animal health has traditionally been defined as the absence of disease and the normal functioning of the organism, including the ability to maintain normal behaviour and cope with environmental disturbances (23, 24). Over time, however, this concept has evolved toward a more holistic perspective that increasingly integrates animal welfare. Animal welfare is commonly framed through the Five Freedoms: freedom from hunger, thirst and malnutrition; freedom from physical discomfort; freedom from pain, injury and disease; the opportunity to express normal species-specific behaviour; and freedom from fear and distress (25). More recently, the Five Domains framework has further expanded this approach by incorporating nutrition, environment, health, behaviour and mental state as key dimensions of animal welfare (26). In this context, cattle health can serve as an important indicator not only of animal welfare but also of the environmental sustainability and technical and economic performance of farming systems. In addition, animal health directly affects animal production and reproductive efficiency (27–29). In livestock research, these domains are increasingly operationalized through multidimensional indicators including environmental conditions (e.g., housing conditions and freedom of movement), clinical indicators (e.g., lameness, lesions, and body condition), biological indicators (e.g., blood markers of inflammation or negative energy balance), zootechnical indicators (e.g., production, reproduction, and longevity), and behavioural indicators (e.g., activity levels, human–animal interactions, and social behaviour within the herd) (30–32). Health assessments can be conducted at both herd and individual levels. At the herd level, commonly used indicators include mortality rates, culling rates, and incidence of diseases such as mastitis or lameness. At the individual level, indicators are generally based on clinical observations and/or biological measurements such as blood analyses. Recent advances in precision livestock farming (PLF) have facilitated the integration of behavioural, production, physiological, and environmental information for animal health assessment (33–35). However, many studies still focus on a limited set of indicators or a single level of observation (36), and comprehensive approaches integrating environmental, zootechnical, clinical, behavioural, and biological dimensions remain relatively scarce, particularly across contrasting dairy production systems and geographical contexts (37).

Despite growing interest in multidimensional approaches to animal health assessment, comprehensive comparisons of cattle health across contrasting dairy production systems remain limited, particularly across different European contexts. Although PLF studies have considerably advanced the integration of behavioural and physiological monitoring in dairy cattle (38, 39), most have focused on intensive production systems or specific health dimensions rather than providing integrated comparisons across dairy production models. Further research is therefore needed to better understand the complex interactions between dairy cow health and farming system design. The present study contributes to this field through an assessment conducted between 2018 and 2022 across diverse European dairy farming conditions. The aim of this study was to assess the health status of dairy cattle across a gradient of production intensification dairy farming systems. Specifically, we compared three systems that coexist in Europe: Low-input grazing systems considered as agroecological system, grazing systems with higher levels of inputs and production, and intensive indoor systems. To achieve this objective, we analyzed different components of animal health (environmental, zootechnical, biological, and clinical) across three complementary levels: herd-level indicators, individual clinical observations, and individual biological measurements. Particular attention was given to integrating indicators collected at different organizational levels in order to provide a comprehensive characterization of cattle health across contrasted dairy production systems.

## 2 Materials and methods

### 2.1 Farm selection

**Figure 1:**
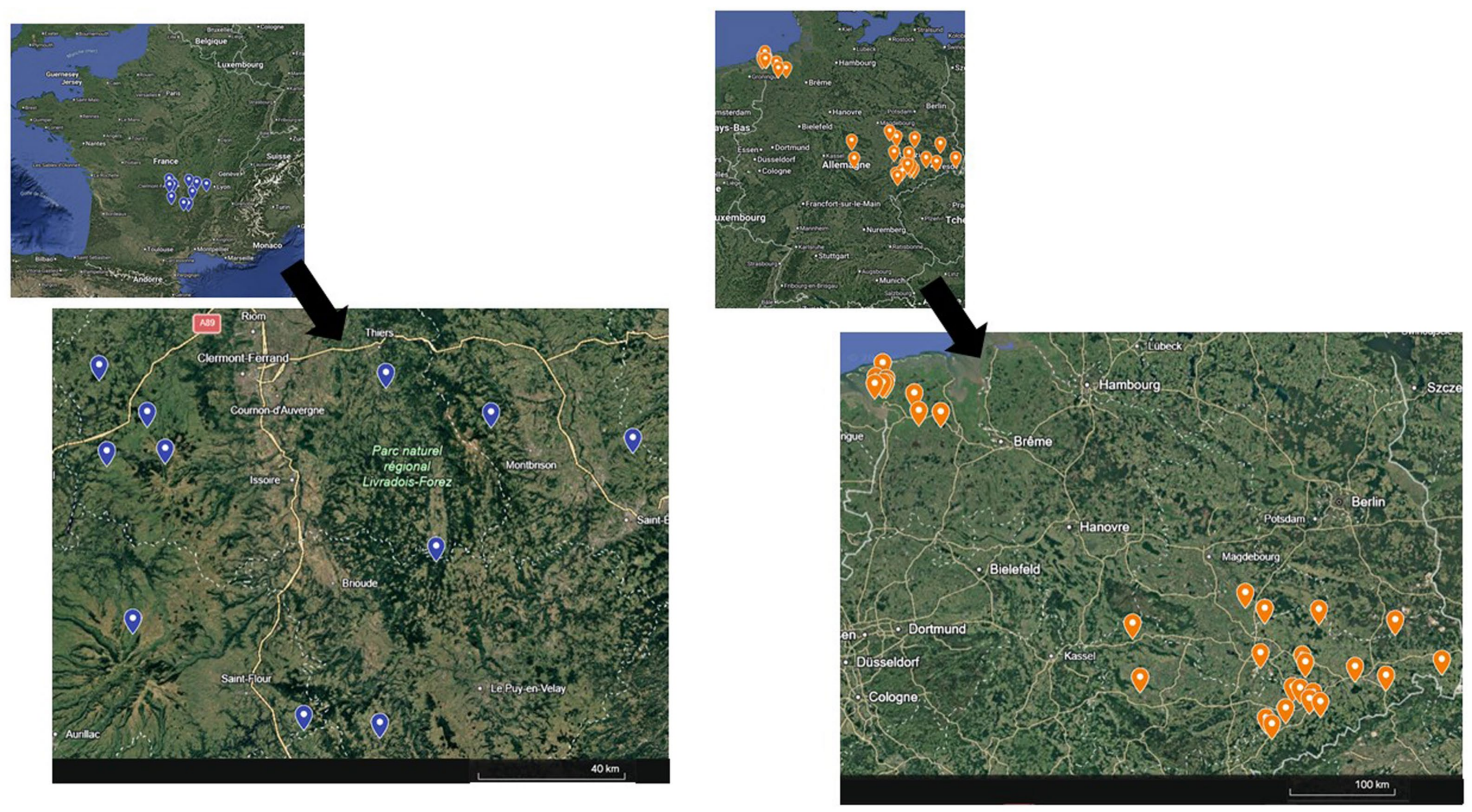
Farms localization. Low-Input farms in blue. GraCow and Indoor farms in orange.

The experiment was conducted in France and Germany in order to capture a broad and biologically meaningful gradient of dairy production intensification under commercial farming conditions. Rather than implementing a fully standardized experimental design, the objective was to characterize health indicators within existing production systems representative of contrasting European dairy models. The study therefore followed an observational and comparative approach based on real-world farming conditions. In Germany, 30 farms mainly located in three federal states: Saxony-Anhalt, Saxony and lower-Saxony were followed from November 20, 2018, to September 24, 2020. In France 11 farms located in the central Massif (Auvergne-Rhone-Alpes) region were followed from January 2021 to July 2022 (Figure 1).

Farm inclusion criteria were defined as follows: (i) commercial dairy farms with a stable herd structure during the observation period; (ii) availability of complete feeding and management records and (iii) willingness to participate over at least one production year. The sampling strategy was specifically designed to exploit the natural diversity of dairy farming systems found in the two countries. Together, these regions encompass a wide range of management strategies, from small-scale agroecological grass-based systems to highly specialized intensive dairy farms. In the central Massif, dairy farms are typically characterized by a strong reliance on permanent grasslands, low external inputs, and moderate milk production. In contrast, northern and eastern Germany are dominated by larger and more specialized farms, including both intensive grazing systems and fully indoor systems relying more heavily on maize silage and purchased concentrates. Combining these contrasting contexts enabled the construction of a relevant intensification gradient that would have been difficult to reproduce within a single country. The purpose of this gradient was not to compare France and Germany as such, but to evaluate dairy production systems differing in their dependence on grassland resources, use of external inputs, herd size, and production intensity. Country was therefore considered as a source of structural diversity that facilitated the identification of contrasting production models. The unequal number of farms between countries reflects structural differences in dairy production systems. French low-input agroecological farms are generally smaller and more heterogeneous, whereas German dairy farms tend to be larger and more specialized. Therefore, the sampling strategy was designed to maximize the representation of contrasting production systems rather than to achieve balanced sample sizes between countries.

Based on criteria related to grassland proportion in heifer or cow feeding and milk production level, an intensification gradient of dairy farming systems was defined in the sample, comprising three categories.

(i) Low-input grazing systems without maize silage and with limited external inputs (Low-input). These farms were primarily grassland-based [grassland area >70% of utilized agricultural area (UAA)], with dairy cows grazing for more than 180 days per year and relatively low milk production levels (<7,000 kg per 305-day lactation; median 4,500 kg). Cows were housed indoors during winter (approximately during 5–6 months, depending on weather conditions and altitude) and grazed during the remainder of the year. Winter housing mainly consisted of either cubicle housing systems or straw-bedded loose housing systems, depending on the farm. These farms are located in the Massif central region of France;
(ii) Conventional grazing systems using maize silage, where heifers and cows have access to pasture (GraCow). These farms are located in Germany and target a milk production of approximately 10,000 kg per 305-day lactation. GraCow farms combined a winter indoor housing period with a grazing season during spring and summer. During the grazing season, lactating cows had regular access to pasture, whereas during winter they were housed indoors;
(iii) Indoor systems characterized by higher production levels and the use of maize silage (Indoor). Indoor farms housed cows indoors throughout the year and did not provide routine access to pasture. These farms are located in Germany and target a milk production of approximately 10,000 kg per 305-day lactation.

Among the 41 sampled dairy farms, 11 belonged to the Low-input grazing systems, 10 to the conventional grazing dairy systems, and 20 to the indoor systems.

On each farm, health was assessed at three levels: farm/herd, individual animal, and blood metabolite analyses, in order to address the different domains of animal welfare, with a particular focus on the health dimension (absence of injuries, and disease). This multiscale approach was chosen because health and welfare are multifactorial and cannot be adequately characterized by a single indicator. Farm-level descriptors provide information on management and housing conditions, animal observations capture clinically visible outcomes, and blood metabolites reflect underlying physiological and metabolic processes related to nutrition, inflammation, and mineral balance.

General characteristics of the production systems were collected at the beginning of the study. The timing and frequency of subsequent measurements differed among production systems according to the objectives and logistical constraints of each study component (Table 1). For the two pasture-based systems (Low-input and GraCow), measurements were conducted during both winter (indoor housing and feeding period) and summer (grazing period) in order to capture seasonal variability in management and diet composition. In Low-input farms, visits were performed between February 2021 and July 2022, resulting in repeated observations spanning two production years for most farms. In GraCow farms, measurements were conducted twice during 2020, once during the indoor winter period (early 2020) and once during the grazing season (summer). For Indoor systems, one measurement per farm was performed (winter 2018–2019 or winter 2019–2020) because these farms were managed under relatively constant feeding and housing conditions throughout the year. As cows remained indoors year-round and management practices varied little seasonally, repeated measurements were not considered necessary. When multiple observations were available for a farm (Low-input systems), all measurements were retained and subsequently averaged at farm level before statistical analyses in order to ensure comparability among production systems.

**Table 1:** Overview of the study design and sampling strategy across production systems.

Production system	Low-input	GraCow	Indoor
Country	France	Germany	Germany
Number of farms	11	10	20
Study period	February 2021 – July 2022	2020	Winter 2018–2019 or Winter 2019–2020
Seasons covered	Winter and grazing	Winter and grazing	Winter
N° of farm visits	4	2	1
N° of farm level assessments and general characteristics	2	2	1
N° of cows assessed per visit	All cows	10 cows	10 cows
Blood samples collected per visit	10% and > 5 cows	10 cows	10 cows
Milk sampling	4 pooled bulk tank	2 pooled herd	2 pooled herd

A summary of the timing and frequency of measurements performed in each production system is provided in Table 1.

### 2.2 Data collection at farm level

#### 2.2.1 General characteristics of the farming systems

Farm-level data were collected on all participating farms. These data included information on farm structure, production, herd management, reproduction, health, and housing conditions (Table 2). Housing type and the level of freedom of movement for cows were also recorded (free-stall or permanent tethering, access to exercise or pasture). “Access to exercise” refers to access to an outdoor exercise area (e.g., loafing yard or paddock) providing outdoor space for movement but without the intention of feed intake, in contrast to pasture access where cows actively graze and ingest a substantial proportion of their diet from grass.

**Table 2:** Indicators used for farm description.

Category	Indicators
Structure	number of employees number of livestock units (LUs) production system (organic or conventional)
Production	milk yield (kg milk/cow/year) milk quality: fat and protein content somatic cell count (SCC)
Herd Management and reproduction	breed age at first calving replacement rate and culling rate calving interval
Health	cow mortality rate mortality rate of calves <1 year old
Housing	housing system (straw-bedded, cubicle, or tethered housing) cow mobility/ease of movement

In Low-input farms, data were primarily extracted from herd management software records maintained on farm. In GraCow and Indoor systems, data were obtained from official milk recording and associated herd performance databases.

Farm-level indicators were derived from annual records routinely collected within each monitoring system. For GraCow and Indoor farms, values corresponded to annual means calculated over the monitored production year. For Low-input farms, which were followed over two production years, values were averaged across the entire monitoring period to obtain a single representative value per farm. Because the study combined datasets collected in two countries within ongoing research programs, some differences in sampling and laboratory procedures were unavoidable. However, all protocols were based on comparable physiological principles and standardized veterinary assessment methods.

To ensure comparability across systems, all farm-level indicators (e.g., milk yield, mortality rates, culling/replacement rates) were expressed at the farm level using a single farm-level value corresponding to the mean over the available monitoring period (1 year for GraCow and Indoor farms, and 2 years when available for Low-input farms), regardless of data source (software records, official recording systems).

#### 2.2.2 Milk sampling and milk quality analysis

In Low-input production systems milk production data were collected at the herd level. Two pooled bulk tank samples per year were collected (one during the grazing period and one during winter, 4 milk samples in total). Samples were preserved with 2-bromo-2-nitropropane-1,3-diol (Bronopol; D&F Inc., Dublin, CA, United States) and stored at 4 °C until analysis. Fat and protein contents were determined by mid-infrared spectroscopy (Agrolab’s, Aurillac, France) according to ISO 9622:2013, and somatic cell count (SCC) was determined by epifluorescence according to ISO 13366-2:2006. Bronopol was used as preservative in the three production systems; in GraCow and Indoor production systems, the preservative additionally contained Kathon as an antimicrobial stabilizer. Both preservative systems are routinely used in official milk recording schemes and are considered compatible with mid-infrared spectroscopy analyses, with no expected interference on milk composition traits measured in this study.

In GraCow and Indoor production systems, milk production was recorded electronically after milking. Composite milk samples were collected once per year in Indoor systems and twice per year in GraCow system (summer and winter). Samples were obtained from a single milking session using the standard milk meters employed for official milk recording by regional herdbook associations (Landeskontrollverbände, LKV). From ten individual cow samples, a pooled farm-level sample was created. The pooled sample was subsequently divided for laboratory analysis. Specifically, 40 mL of milk were transferred into LKV barcode tubes and sent to LKV Mecklenburg-Vorpommern, where samples were cooled and preserved with a Bronopol-based preservative solution supplemented with Kathon antimicrobial stabilizer. In addition to pooled samples, individual milk samples (40 mL per animal) were also provided to the laboratory. Milk samples were analyzed by the Milchkontroll- und Rinderzuchtverband eG laboratory (Güstrow, Germany), an accredited testing laboratory (D-PL-19638-01-00) according to DIN EN ISO/IEC 17025:2018. Milk protein, fat, and lactose contents were determined using infrared spectrophotometry (MilkoScan FT6000, Foss GmbH, Hamburg, Germany), while SCC was measured using a fluorescence-optical counting system (Fossomatic FC, Foss GmbH). Mid-infrared spectroscopy (MIRS) was additionally used to determine milk fatty acid composition.

### 2.3 Data collection at animal level

In Low-input farms, herd size was sufficiently small to allow assessment of all lactating cows. In contrast, GraCow and Indoor farms were substantially larger with mean (± SD) herd sizes of 158 ± 124 and 444 ± 272 livestock units, respectively, making exhaustive individual evaluation impractical. Therefore, a targeted subsampling strategy was applied. In GraCow and Indoor production systems, ten cows per farm were selected according to predefined criteria: cows were required to be in second or greater lactation and beyond 100 days in milk (DIM). This strategy aimed to reduce variability associated with early lactation and primiparous animals. This approach allowed the collection of standardized physiological and clinical information while maintaining feasibility under commercial farm conditions.

#### 2.3.1 Animal observation

Cows were observed to assess body condition score (BCS), lameness, and cleanliness. In Low-input and GraCow production systems, cows were observed twice per year (winter and summer), whereas Indoor cows were observed once per year. The same cows were followed and used for all measurements within a given observation period. However, repeated observations did not necessarily involve the same individuals across seasons in all farms. Herd-level averages were calculated for each visit before aggregation across seasons and years. In addition, some methodological differences existed between production systems because data collection originated from two independent but complementary research programs. However, all protocols relied on comparable physiological principles and internationally recognized veterinary assessment approaches. Body condition score, lameness, and cleanliness assessments were conducted using validated scoring systems based on ordinal five-point scales.

##### 2.3.1.1 Body condition score

In Low-input production systems, all cows were scored for body condition by trained assessors using the scale developed by Bazin (40). In GraCow and Indoor production systems, scoring followed the protocol described by Edmonson et al. (41). Although different scoring protocols were used in the three production systems, both protocols use a five-point scale with increments of 0.25, ranging from 1 (or 0) = emaciated to 5 = obese. Optimal Body Condition Score (BCS) values for dairy cows generally range between 2.5 and 3.5 depending on the stage of lactation. Both scoring was based on visual and tactile assessment of fat cover over the loin, ribs, and tail head areas, and was performed by trained observers to ensure scoring consistency. Each cow was assessed individually under similar lighting and standing conditions.

##### 2.3.1.2 Lameness

Lameness was assessed using the “Médecine de troupeau en élevage laitier” (herd health in dairy farming) protocol in Low-input production systems (42) and the method of Sprecher et al. (43), adapted by Starke et al. (44) and Ebert et al. (45), in GraCow and Indoor production systems. Both methods use a five-point scoring system, which is comparable between the two approaches. Cows were observed walking in a straight line on a non-slippery walking alleys routinely used for animal handling on each farm. Although flooring material was not fully standardized across farms, observations were conducted under comparable walking conditions to minimize environmental effects on gait assessment. Cows were scored as follows:

1 = normal locomotion;

2 = mild gait irregularity (slightly hunched back when walking but flat when resting/standing).

3 = moderate lameness, including shortened strides or arched back (when walking and when resting/standing).

4 = severe lameness, with marked asymmetry and reluctance to bear weight.

5 = extreme lameness, with very limited weight-bearing and evident pain behaviour.

##### 2.3.1.3 Cleanliness

Cleanliness was assessed using the “Médecine de troupeau en élevage laitier” (herd health in dairy farming) protocol (42) in Low-input production systems and the protocol described by Reneau et al. (46) in GraCow and Indoor production systems. The two comparable methods use a five-point scoring scale, where 1 indicates a clean animal (no visible dirt) and 5 indicates a very dirty animal (plaque of dirty >50% area). In both protocol, cleanliness is scored visually on three body regions: udder, lower hind legs, and flank areas. Both protocols evaluate similar body regions and use equivalent ordinal scales, allowing comparison at herd level despite minor methodological differences.

#### 2.3.2 Blood sampling

Blood samples were collected from the coccygeal vein in all production systems using vacuum blood collection systems containing anticoagulant or serum collection tubes. In Low-input production systems, EDTA vacuum tubes (Terumo France, Guyancourt, France) were used, whereas in GraCow and Indoor production systems, 10 mL Kabevette® collection tubes (Kabe Labortechnik GmbH, Germany) were used according to local laboratory procedures. Samples were centrifuged immediately after collection using standardized laboratory procedures specific to each laboratory (France: 1200 × *g* for 20 min at 4 °C; Germany: 1100 × *g* for 10 min at 20–22 °C). Plasma or serum aliquots were stored at −20 °C until analysis.

Although minor methodological differences existed between laboratories, all procedures followed validated veterinary diagnostic protocols routinely used for metabolite analysis in dairy cattle. The metabolites investigated in this study are considered stable under the centrifugation and storage conditions applied. Therefore, no major analytical bias related to sample handling was expected. Blood analyses were performed to quantify metabolites reflecting the nutritional status, inflammatory status, and neuromuscular function of the animals (Table 3).

**Table 3:** Description of the parameters measured, their reference values and their implications for health.

Parameters	Units	Reference range	Health interpretation	Notes on abnormal values	Reference citation
NEFA (Non-Esterified Fatty Acids)	μmol/L	<150 antepartum <600 postpartum	Energy balance	Elevated indicates negative energy balance; risk of subclinical ketosis	(60, 61)
BHB (β-Hydroxybutyrate)	mmol/L	0.3–0.7	Energy balance	>1 mmol/L indicates negative energy balance and risk of subclinical ketosis	(63)
Urea	mmol/L	2.5–6.5	Reflects dietary protein balance and liver function	>7–8 mmol/L indicates energy imbalance/hepatic stress	(64, 65)
Total protein	g/L	60–80	Reflects nutritional, inflammatory, and immune status	Valuers out of range indicates dietary imbalance, kidney/digestive pathology	(64, 65)
Albumin	g/L	30–40	Indicates nutritional and liver health	<30 g/L indicates inflammation, chronic disease, metabolic stress	(66)
Calcium	mmol/L	2.0–2.5	Calcium balance (muscle, nerve function)	Low values indicate hypocalcemia, reproductive issues, milk fever	66
Phosphorus	mmol/L	1.4–2.3	Essential for metabolism and bone mineralization	Values out of range indicates immunosuppression, reproductive/productivity issues	(65)
Magnesium	mmol/L	0.75–0.8	Required for energy metabolism, muscle and immune function	<0.75 mmol/L indicates oxidative stress, reproductive/productivity disorders	(65)
Sodium	mmol/L	135–150	Maintains water balance, nerve and muscle function	<135 mmol/L indicates metabolic alkalosis, renal disorders, decreased productivity	(68)
Potassium	mmol/L	3.9–5.2	Maintains electrolyte and acid–base balance, ensure proper muscle, nerve, and cardiac functions	Low values impar muscle contraction and nerve function	(68)

In Low-input production systems, blood samples were collected from approximately 20% of animals per farm, with a minimum of five cows per farm. Biochemical analyses were performed using an Arena 20 XT Chemistry System (Thermo Scientific, Waltham, MA, United Sates). Concentrations of non-esterified fatty acids (NEFA), *β*-hydroxybutyrate (BHB), urea, total protein, and albumin were determined according to the manufacturers’ instructions. Plasma sodium (Na^+^), potassium (K^+^), and chloride (Cl^−^) concentrations were measured using ion-selective electrodes (ISE).

GraCow and Indor production systems serum samples were analyzed using a Cobas c311 system (Roche Diagnostics, Mannheim, Germany) for a clinical chemistry panel including calcium, magnesium, sodium, potassium, total protein, urea, albumin, and *β*-hydroxybutyrate. Non-Esterified Fatty Acids (NEFA) concentrations were determined using a colorimetric assay (Randox Laboratories Ltd.).

Analyses were conducted in accredited veterinary diagnostic laboratories using validated and automated systems routinely employed for bovine metabolic profiling. Although no formal inter-laboratory calibration study was performed, comparable analytical principles (enzymatic colorimetric assays and ion-selective electrodes), certified quality-control procedures were applied. The veterinary laboratory follows a laboratory quality management program including daily quality controls and a trimestral external quality program assessment according to ASVCP guideline (external quality assessment and comparative testing for reference in clinic laboratories) (47, 48). These proficiency testing programs provide continuous external benchmarking and ensure the robustness and comparability of analytical results across laboratories. While no direct inter-laboratory calibration was performed, these established quality assurance procedures are widely recognized as effective in minimizing systematic analytical bias in multi-laboratory studies.

### 2.4 Ethics approval

All procedures complied with national regulations and institutional guidelines governing the use of animals for scientific purposes and animal welfare. In France, the study received approval from the VetAgro Sup Ethical Committee under authorization number 2086. In Germany, blood samples were collected exclusively within the framework of routine metabolic monitoring and regular herd health management practices performed in the participating dairy herds by veterinary clinicians.

### 2.5 Data analysis

Individual animal data (observational scores and blood parameters) were averaged at the herd level prior to statistical analysis. When multiple measurements were available across seasons and/or years, these were first aggregated within farm to obtain a single mean value per variable over the full monitoring period. Thus, each farm contributed one final value per variable to the dataset used for statistical analyses. Accordingly, the farm was considered the experimental unit, and all farms contributed equally to the analysis regardless of herd size or number of repeated measurements. No weighting factor was applied, as the experimental unit considered in the analyses was the farm rather than the individual cow. This approach ensured equal contribution of each farm to the comparison between production systems. Clinical, and biological variables were aggregated at herd level before analysis. Milk yield data used in statistical analysis was obtained from routine herd recording systems and expressed as a farm-level mean over the monitored period. In contrast, milk composition variables (fat, protein, lactose, somatic cell count, and fatty acid profile) were derived from the milk samples collected within the framework of this study. Only herd-level aggregated values were used for inter-system comparisons to ensure consistency of the analytical unit across countries.

Statistical analyses were performed using R software (version 4.5.0; R Foundation for Statistical Computing, Vienna, Austria). Data visualization were conducted using RStudio (Posit Software, Boston, MA, United States). Due to the confounding effect between country and production system (Low-input, GraCow, Indoor), only the system factor is retained as a fixed factor. Country could not be included as an independent fixed effect in the statistical models and was treated as a structural component of the production system design. This choice reflects the study objective, which focused on real-world system-level differences rather than isolating country effects. Consequently, only production system was retained as a fixed factor, and country was considered an intrinsic component of the production system definition. Differences among systems were assessed using a one-way analysis of variance (ANOVA) based on the following linear model: y = lm(x ∼ system). Model assumptions were evaluated prior to interpretation. Residual normality and homoscedasticity were checked visually and with Shapiro–Wilk and Levene test prior to model interpretation. When the ANOVA indicated a significant effect of production system, pairwise comparisons among systems were conducted using Tukey-adjusted *post hoc* tests. Statistical significance was considered at *p* < 0.05. For variables with missing data in the GraCow system (e.g., cow mortality rate, calf mortality rate, and culling age), statistical analyses were restricted to comparisons between the Low-input and Indoor systems.

In Tables 4–6, descriptive statistics are presented as raw farm-level means, standard deviations (SD), and 95% confidence intervals (CI) calculated from the observed data within each production system. These values correspond to the aggregated farm-level observations used in the ANOVA and are not model-adjusted estimated marginal means (EMM). In the Results section, only raw means and standard deviations are reported for brevity, whereas the full tables additionally include 95% confidence intervals to reflect the precision of the estimates. Because of the observational nature of the study and the partial confounding between country and production system, statistical associations should not be interpreted as strictly causal relationships.

**Table 4:** Comparison of key herd management variables among the three production systems.

Indicators	Low-input	GraCow	Indoor	*p*-value
Livestock unit (dairy cows)	40.7^a^ +/−20.9; 26,7-54,8	158^b^ +/−124; 69.0–246	453^b^ +/−269; 328–579	**<0.01**
Milk production/cow/year (kg)	4573^a^ +/−1,282; 3,751–5,394	9531^b^ +/−1,040; 8,709–10,352	9965^b^ +/− 1,027; 9,484–10,446	**<0.01**
Age at first calving (month)	32.8^a^ +/−2.38; 31.2–34.3	27.4^b^ +/−1.17; 25.8–29.0	25.7^c^ +/−1.09; 25.1–26.1	**<0.01**

Values are presented as raw mean ± standard deviation, with 95% confidence intervals. Different superscript letters within a row indicate significant pairwise differences between systems after post hoc multiple comparison tests (*p* < 0.05), whereas identical letters indicate no significant difference. Values shown in bold represent statistically significant *p*-values.

**Table 5:** Description of health variables at farm level, across the three systems and the significance of the differences between the three systems.

Indicators	Low-input	GraCow	Indoor	*p*-value
Reproduction				
Calving interval (days)	386^a^ +/−28.7; 366–404	416^b^ +/−13.8; 397–435	413^b^ +/−23.2; 402–424	**0.01**
Mortality				
Cow mortality rate	3.8^a^ +/−4.04; 1.08–6.52	Missing data	9.82^b^ +/−6.06; 6.99–12.8	**<0.01**
Calve death rate	10.27^a^ +/− 10.22; 3.40–17.1	Missing data	7.15^a^ +/− 2.15; 6.14–8.16	0.35
Culling				
Culling age (year)	9.09^a^ +/−1.6; 8.00–10.18	Missing data	4.93^b^ +/−0.34; 4.77–5.09	**<0.01**
Culling rate	21.8^a^ +/−8.36; 16.2–27.4	22.8^a^ +/−5.07; 17.2–28.5	31.8^b^ +/−6.31; 28.8–34.8	**<0.01**
Milk quality				
Fat content (g/kg)	40.2^a^ +/−2.42; 38.56–41.82	40.5^a^ +/−1.01; 38.9–42.2	39.8^a^ +/−1.71; 39.0–40.6	0.61
Protein content (g/kg)	32.8^a^ +/−1.56; 31.8–33.9	34.3^ab^ +/−0.62; 33.3–35.4	34.4^b^ +/−1.42; 33.8–35.1	**0.01**
Somatic cells (cells/ml of milk; log)	5.28^a^ +/−0.2	5.31^a^ +/−0.14	5.31^a^ +/−0.08	0.88

Values are presented as raw mean ± standard deviation, with 95% confidence intervals. Different superscript letters within a row indicate significant pairwise differences between systems after post hoc multiple comparison tests (*p* < 0.05), whereas identical letters indicate no significant difference. For calving interval, no significant difference was observed between GraCow and Indoor systems (pairwise comparison: *p* = 0.91). For culling rate, no significant difference was observed between Low nput and GraCow systems (pairwise comparison: *p* = 0.95). “Missing data” indicates variables not available for the GraCow system and therefore not included in statistical comparisons involving this group. Somatic cell count (SCC) was log10-transformed prior to analysis. Values shown in bold represent statistically significant *p*-values.

**Table 6:** Description of animal observation variables and blood metabolites across the three systems and the significance of the differences between the three systems.

Indicators	Low-input	GraCow	Indoor	*p*-value
Clinical observation				
BCS	3.22^a^ +/− 0.269; 3.04–3.40	2.38^b^ +/−0.104; 2.20–2.56	2.58^b^ +/−0.217; 2.48–2.68	**<0.01**
Lameness score	1.04^a^ +/−0.052; 1.00–1.07	1.48^b^ +/−0.293; 1.45–1.52	1.86^c^ +/−0.480; 1.64–2.09	**<0.01**
Cleanliness score	1.63^a^ +/−0.302; 1.42–1.83	2.06^b^ +/−0.217; 1.85–2.26	2.17^b^ +/−0.427; 1.97–2.37	**<0.01**
Blood				
NEFA (umol/L)	184^a^ +/−117; 106–263	101^ab^ +/−33.3; 22.0–180	99.4^b^ +/−82.4; 60.9–138	**0.04**
BHB (mmol/L)	0.587^a^ +/− 0.158; 0.480–0.690	0.790^b^ +/−0.120; 0.680–0.900	0.752^b^ +/−0.188; 0.660–0.840	**0.02**
Urea (mmol/L)	3.55^a^ +/−0.99; 2.88–4.22	4.18^a^ +/−0.447; 3.51–4.85	3.68^a^ +/−0.899; 3.26–4.10	0.23
Protein g/L	74.1^a^ +/−4.84; 70.9–77.4	76.0^a^ +/−1.97; 72.7–79.2	77.1^a^ +/−2.82; 75.7–78.4	0.09
Albumin g/L	31.1^a^ +/−1.82; 29.8–32.3	39.5^b^ +/−1.12; 38.3–40.7	39.5^b^ +/−1.86; 38.7–40.4	**<0.01**
Calcium (mmol/L)	2.22^a^ +/−0.242; 2.06–2.38	2.39^b^ +/−0.042; 2.23–2.56	2.47^b^ +/−0.072; 2.44–2.50	**<0.01**
Phosphorus (mmol/L)	1.78^a^ +/−0.151; 1.68–1.89	1.77^a^ +/−0.099; 1.67–1.87	1.78^a^ +/−0.114; 1.73–1.83	0.95
Magnesium (mmol/L)	0.841^a^ +/−0.094; 0.780–0.900	0.975^b^ +/−0.035; 0.91–1.94	0.990^b^ +/−0.045; 0.97–1.01	**<0.01**
Sodium (mmol/L)	142^a^ +/−15.8; 131–152	139^a^ +/−1.12; 129–150	139^a^ +/−2.49; 138–140	0.65
Potassium (mmol/L)	4.44^a^ +/−0.179; 4.32–4.56	4.39^a^ +/−0.121; 4.27–4.51	4.49^a^ +/−0.231; 4.38–4.60	0.38

Values are presented as raw mean ± standard deviation, with 95% confidence intervals. Different superscript letters within a row indicate significant pairwise differences between systems after post hoc multiple comparison tests (*p* < 0.05), whereas identical letters indicate no significant difference. Values shown in bold represent statistically significant *p*-values.

## 3 Results

### 3.1 Farms description

Significant differences were observed among Low-input, GraCow, and Indoor production systems for the structural and zootechnical-productive dimensions (Table 4). Livestock units (LU) and milk production per cow in Low-input production systems (40.7 LU and 4,573 kg milk/cow/year) were significantly lower than in the two other production systems (158 and 453 LU, respectively; 9,531 kg and 9,965 kg milk/cow/year for GraCow and Indoor production systems, respectively; *p* < 0.001).

The cow breeds identified in the Low-input production system were highly diverse, including both high-yielding breeds (such as Prim’Holstein and Montbéliarde) and less productive or local breeds (e.g., Ferrandaise, Vosgienne). In addition, six farms within this system kept more than one breed. In contrast, the two other systems were composed exclusively of Holstein cows, with the exception of one Indoor farm that combined Holstein and Fleckvieh breeds. The Low-input grazing system was predominantly composed of organically certified farming, with nine out of eleven farms holding organic certification. Moreover, six Low-input farms carried out on-farm cheese processing, producing protected designation of origin (PDO) cheeses. With respect to the environmental dimension, particularly freedom of movement, cows in the GraCow and Indoor systems were housed in loose housing systems allowing unrestricted movement. In the Low-input system, 4 out of 11 farms still used tie-stall housing, in which animals were tethered for part of the year. These characteristics were included as descriptive variables to better characterize the diversity of the Low-input grazing system and were not directly included in the statistical analysis of system-level differences.

### 3.2 Health at farm level

Zootechnical and reproductive performance differed between systems. Calving interval was shorter in Low-input systems (386 days) compared with GraCow (416 days) and Indoor systems (413 days), whereas no significant difference was observed between GraCow and Indoor production systems (*p* = 0.91) (Table 5). Regarding mortality, Indoor production systems exhibited higher cow mortality rates than Low-input systems (*p* < 0.01). As data were unavailable for GraCow production systems, this comparison was limited to Low-input and Indoor production systems. Similarly, no significant differences were observed in calf mortality between Low-input and Indoor production systems (*p* = 0.35).

Age at first calving differed significantly among production systems, with higher values in Low-input production systems (32.8 months) compared with GraCow (27.4 months) and Indoor production systems (25.7 months) (Table 3). Indoor production systems also exhibited a higher culling rate (31.8%) than the other production systems, whereas no significant difference was observed between Low-input and GraCow systems (*p* = 0.95). In addition, cows in Low-input production systems had a higher culling age than those in Indoor production systems (*p* < 0.01). As culling age data were unavailable for GraCow systems, this comparison was restricted to Low-input and Indoor systems.

Milk quality indicators differed slightly among production systems. Milk fat content did not differ among production systems (*p* = 0.61). In contrast, protein concentration was lower in Low-input production systems (32.8 g/kg) and highest in Indoor systems (34.4 g/kg). GraCow herds had intermediate protein concentrations (34.3 g/kg) that did not differ significantly from either Low-input or Indoor production systems (*p* = 0.014). Somatic cell count was similar across systems (*p* = 0.88).

### 3.3 Animal health observation

Some clinical indicators differed significantly among production system such as BCS (*p* < 0.01). Cows from Low-input production systems showed higher BCS (3.22) than those from the GraCow (2.38) and Indoor production systems (2.58), whereas no significant difference was observed between the latter two production systems (Table 5). Lameness scores also differed significantly among systems (*p* < 0.01), with lower scores in the Low-input system (1.04) compared with both the GraCow (1.48) and Indoor production systems (1.86). Cleanliness scores were significantly lower in the Low-input production system than in the other two systems (*p* < 0.01), reflecting better overall animal cleanliness.

Regarding biological indicators and nutritional status, and more specifically energy balance, cows in the Low-input production system exhibited higher NEFA concentrations (184 μmol/L) compared with those in the Indoor system (99.4 μmol/L) (*p* = 0.035), while the GraCow production system showed intermediate values (101 μmol/L) that did not differ significantly from either group (Table 5). Although these values differed statistically, NEFA concentrations in all production systems remained within the physiological reference range (Table 3). *β*-hydroxybutyrate (BHB) concentrations were lower in the Low-input system (*p* = 0.02) than in the GraCow and Indoor production systems, However, BHB values in GraCow and Indoor production systems were above the upper reference threshold for healthy animals, particularly after the peak of lactation, but remained below the ketosis threshold (1.2 mmol/L) reported in Table 3. No significant differences were observed among systems for urea concentrations (*p* = 0.23), suggesting similar protein nutritional balance. Likewise, total protein concentrations, reflecting both nutritional and inflammatory status, did not differ significantly between systems (*p* = 0.09). Albumin concentrations differed significantly among production systems (*p* < 0.01), with lower values observed in the Low-input production system (31.1 mmol/L) compared with both the GraCow (39.5 mmol/L) and Indoor production systems (39.5 mmol/L).

With regard to minerals, as deficiencies can affect productivity, reproduction and nervous and muscular metabolism, no significant differences were found for phosphorus, sodium, or potassium (*p* > 0.05). Significant differences were observed for calcium (*p* < 0.01) and magnesium (*p* < 0.01) concentrations. Both minerals were lower in the Low-input production system (2.22 mmol/L and 0.841 mmol/L) than in the GraCow (2.39 mmol/L and 0.975 mmol/L) and Indoor production systems (2.47 mmol/L and 0.990 mmol/L).

## 4 Discussion

### 4.1 Study limitations

Several limitations should be considered when interpreting the present results. Due to the observational design of the study, the reported differences should be interpreted as system-associated patterns rather than causal relationships. In addition, production system and country were partially confounded, as all Low-input farms were located in France whereas GraCow and Indoor farms were located in Germany. Consequently, observed differences may also reflect country-specific factors, including breed composition, management practices, sampling procedures, and laboratory analyses. Production systems also differed simultaneously in several structural and management characteristics (e.g., feeding strategy, herd size, housing, and milk production level), which could not be statistically disentangled. Furthermore, differences in animal selection criteria, biological matrices, and analytical procedures between countries may have affected the comparability of some metabolic indicators.

These limitations justify interpreting the observed differences as associations characterizing real-world dairy production systems rather than as direct causal consequences of production-system intensity.

### 4.2 Farming systems gradient and structural differences

This study was conducted across a multidimensional gradient of dairy production intensification in two European countries where the dairy sector represents an important component of the national agricultural economy (49). Rather than using a strictly controlled experimental design, the study aimed to characterize health, metabolic, and productive indicators under commercial farming conditions representative of the diversity of European dairy systems.

The intensification gradient was primarily defined using milk production level, feeding strategy, which are widely recognized indicators of dairy system intensification in the literature (50–52). Indeed, the Low-input production system relied mainly on conserved grass and grazing, the GraCow production system combined grazing with maize silage supplementation, whereas the Indoor system was based on year-round indoor feeding with conserved grass and maize silage. In addition, structural characteristics commonly associated with intensification also differed between systems, including herd size and breed composition (11, 53). Low-input production systems were generally characterized by smaller herd sizes (41 cows in mean, from 18 to 80) and greater breed diversity, including local breeds with lower milk production potential, whereas GraCow (158 LU in mean, from 78 to 509) and Indoor (453 LU in mean, from 88 to 1,001) production systems were dominated by Holstein cows - highly specialized dairy breeds. Consequently, differences in milk yield observed between systems likely reflected the combined effects of feeding strategy, production intensity, and genetic potential rather than management practices alone.

Taken together, the combination of herd structure, feeding strategies, housing, production levels, and breed specialization confirms that the farms included in this study represent a multidimensional, although not strictly controlled, intensification gradient, consistent with the diversity of dairy systems described across Europe (8, 11). However, because these dimensions co-varied between systems, their individual contributions could not be disentangled within the present observational design. Furthermore, the imbalance between countries, breeds, and production systems remains an important limitation, meaning that the observed differences should be interpreted as system-associated patterns influenced by both production-system characteristics and country-specific contexts rather than as strictly causal effects of intensification alone.

### 4.3 Health indicators, feeding and productive performances

At the herd level, several indicators suggested differences in health and production dynamics between systems. Low-input production systems were characterized by lower milk yield per cow, higher BCS, and distinct metabolic profiles compared with GraCow and Indoor production systems, consistent with their lower level of production intensification. Despite these differences, most milk and blood biomarkers remained within established physiological reference ranges, suggesting that cows across all systems maintained overall metabolic homeostasis and no evidence of clinically relevant metabolic dysfunction. These reference intervals were compiled from peer-reviewed literature and standard veterinary clinical chemistry references (Table 3). However, they should be interpreted as indicative rather than absolute thresholds, as reference values may vary according to breed, physiological stage, analytical method, biological matrix, and geographical context.

Milk quality indicators, showed contrasting patterns depending on the parameter considered. SCC, a widely used indicator of udder inflammation and intramammary infection, did not differ significantly between systems and remained relatively stable at approximately 200,000–210,000 cells/mL. Although values remained below the European regulatory threshold for non-compliant milk (400,000 cells/mL), they were close to commonly used cut-offs for subclinical mastitis [~200,000 cells/mL, (54, 55)] Importantly, SCC values remained within ranges generally considered compatible with acceptable udder health at herd level, suggesting similar inflammatory status across production systems without clinically meaningful differences. Nevertheless, SCC interpretation should account for potential differences in housing hygiene, milking procedures, bedding conditions, and herd management practices between production systems and countries (56).

Milk fat and protein contents are commonly used as indirect indicators of nutritional and metabolic status in dairy cows because they reflect rumen fermentation patterns, energy balance, and nutrient utilization (57). Milk fat concentration did not differ between production systems, whereas milk protein concentration was slightly lower in Low-input production systems, although all values remained within physiological ranges (≈31–35 g/kg). The lower protein concentration observed in Low-input production systems likely reflects lower dietary energy density and reduced glucogenic precursor supply associated with forage-based diets, which may limit microbial protein synthesis and amino acid availability for milk protein production (58, 59). In contrast, the fat-to-protein ratio remained remarkably stable across production systems (1.18–1.22), well below thresholds commonly associated with severe negative energy balance (>1.35–1.50) or subacute ruminal acidosis (<1.0) (57, 60). The stability of this ratio therefore supports the interpretation that ruminal fermentation and overall energy metabolism remained physiologically balanced across production systems despite differences in production intensity.

Blood metabolite analyses provided further insights into the metabolic and nutritional status of cows across production systems. During early and peak lactation, cows commonly experience negative energy balance, due to energy demands exceeding dietary intake, resulting in adipose tissue mobilization and increased circulating NEFA (60, 61). Accordingly, NEFA concentrations were higher in Low-input production systems, indicating greater lipid mobilization. However, values remained within commonly reported postpartum ranges (approximately <0.5–0.6 mmol/L), suggesting moderate rather than severe metabolic stress (62, 63). In contrast, BHB concentrations were lower in Low-input production systems than in the other systems. This apparent discrepancy may reflect differences in how mobilized fat was metabolized by the liver. NEFA released from adipose tissue can either be fully oxidized to produce energy or converted into ketone bodies such as BHB when hepatic metabolic capacity is exceeded (64). Therefore, the combination of higher NEFA but lower BHB concentrations in Low-input production systems may indicate that cows were mobilizing body reserves while still maintaining efficient hepatic energy metabolism, thereby limiting ketone body production. Although BHB concentrations in GraCow and Indoor production systems occasionally exceeded values considered optimal for mid- to late-lactation cows, all measured values remained below commonly accepted thresholds for subclinical ketosis (>1.2–1.4 mmol/L) (65, 66), indicating no evidence of clinically relevant ketotic disorders. Importantly, cows included in this study were generally beyond peak lactation, a stage at which metabolic pressure is lower than during early lactation. Consequently, the observed NEFA and BHB concentrations should be interpreted as indicators of metabolic adaptation to different production systems rather than markers of severe metabolic stress or clinical disorders. Interpretation is also limited by differences in animal selection criteria between countries. In German farms, cows were selected according to predefined criteria (≥ second lactation and >100 DIM), whereas Low-input farms included a broader representation of lactating cows. Differences in parity and stage of lactation may therefore have contributed to part of the observed variation in metabolic indicators.

Other blood metabolites also supported the interpretation of globally preserved metabolic function across systems. Blood urea concentrations did not differ significantly between production systems and remained within commonly accepted physiological ranges for lactating cows (approximately 2–6 mmol/L), reflecting similar protein–energy balance and nitrogen utilization efficiency across feeding strategies (67, 68). Likewise, total protein concentrations were stable and within reference limits (approximately 65–80 g/L), suggesting maintained systemic protein metabolism and no evidence of major dehydration or inflammatory processes, although these ranges vary with breed, lactation stage, and management conditions (67, 68).

Albumin concentrations were significantly lower in Low-input systems, but values still fell within physiological intervals for lactating dairy cows (approximately 30–40 g/L) (69). As a marker of hepatic protein synthesis influenced by nutritional status, inflammation, hydration, and physiological stage (70), albumin would be expected to decrease in the presence of marked liver dysfunction or systemic inflammation. Given that urea and total protein remained stable across systems, the lower albumin levels observed in Low-input herds are unlikely to indicate clinically relevant hepatic impairment or inflammatory disease. They more likely reflect subtle differences in dietary protein concentration, energy supply, body reserve dynamics, or breed-related physiological traits typical of forage-based systems. However, interpretation of total protein and albumin concentrations should be made cautiously because analyses were conducted using different biological matrices and laboratory platforms across countries (plasma in France versus serum in Germany). Since serum and plasma protein concentrations are not strictly equivalent due to coagulation-related proteins, part of the observed variation may reflect methodological rather than biological differences.

Electrolyte profiles further confirmed the overall metabolic stability of cows in all systems. Sodium and potassium concentrations remained within physiological ranges (approximately 139–149 mmol/L for Na and 4.3–5.2 mmol/L for K) and did not differ significantly between production systems, indicating preserved osmotic and electrolyte homeostasis (71). Because these ions are tightly regulated and usually altered only in the context of severe metabolic or hydration disturbances, their stability supports the absence of major systemic dysfunction.

Clinical observations of body condition score (BCS) revealed significant differences between production systems, with cows in Low-input production systems exhibiting higher BCS. These findings are consistent with previous studies (72, 73) showing that post-calving BCS loss is associated with higher lactation peaks and greater total milk production over the course of lactation. However, interpretation of BCS differences requires caution. BCS is influenced by multiple factors including breed, parity, stage of lactation, and milk production level (72, 73). Furthermore, BCS assessments, milk variables, and blood biomarkers were not necessarily obtained from exactly the same animals within farms, and herd-level averages may therefore integrate different sources of biological variation. Consequently, the higher BCS observed in Low-input systems cannot be attributed solely to production-system characteristics and may partly reflect differences in animal populations among systems. The combination of higher BCS, moderate NEFA concentrations, and lower BHB concentrations observed in Low-input production systems therefore suggests a distinct metabolic strategy characterized by lower production-driven energy expenditure and more moderate mobilization of body reserves. BCS is strongly influenced by breed, parity, and production level, and may partly reflect systemic differences in energy partitioning rather than nutritional advantage. In addition, although a standardized scoring method was used across countries, some observer-related variability cannot be completely excluded.

Overall, despite some statistically significant differences in milk composition, metabolites, and body condition, all indicators remained within physiological ranges. Overall, when interpreted as a metabolic profile encompassing protein metabolism, hepatic synthetic capacity, renal function, and electrolyte balance, these results indicate that cows across production systems remained in a stable physiological state. The absence of changes in related biomarkers and while remaining within physiological reference ranges, suggests limited biological significance and is more likely attributable to subtle nutritional, physiological, or genetic differences rather than clinically relevant metabolic or inflammatory alterations.

### 4.4 Reproductive performances, locomotion and cow longevity

Low-input production systems were associated with shorter calving intervals (391 days) compared with more intensive systems (413–416 days). This finding is consistent with previous studies reporting that selection for high milk production is often associated with reduced reproductive performance and increased culling rates (14, 15). This pattern is largely explained by the metabolic and endocrine consequences of high milk yield. In early lactation, high-producing cows commonly experience negative energy balance (NEB), when energy output exceeds dietary intake (65). This state is associated with increased mobilization of body reserves, elevated NEFA concentrations, and endocrine adaptations including reduced insulin and IGF-1, which together impair ovarian function and delay resumption of cyclicity (74, 75). In contrast, grazing-based production systems generally involve lower production pressure and may therefore favor improved reproductive performance and longer productive lifespans (73). Lower production pressure may reduce the severity and duration of NEB, thereby favoring reproductive recovery and more efficient allocation of nutrients toward reproduction (76). However, longevity remains a multifactorial trait influenced by management, replacement policies, and breeding strategies, so the higher culling age observed in Low-input production systems should not be interpreted as a purely causal effect of production system (20). Macro-mineral homeostasis contributes to reproductive physiology through its involvement in ovarian activity, follicular development, uterine function, and early embryonic development (77). In this study, calcium and magnesium concentrations were slightly lower in Low-input production systems but remained within physiological ranges (68), whereas phosphorus concentrations did not differ between systems, suggesting no clinically relevant mineral imbalance affecting fertility.

Locomotor health showed clearer differences between systems. Lameness was evaluated using an ordinal locomotion score and lower scores were observed in Low-input production systems. The lower lameness scores observed in these production systems are consistent with previous research highlighting the beneficial effects of grazing on locomotion and hoof health (16, 21). In Europe, lameness prevalence in indoor systems often ranges from 20–40%, compared with 10–12% in pasture-based systems (78). Pasture access improves hoof health by increasing locomotion and reducing exposure to hard flooring (79, 80). However, lameness remains multifactorial and also depends on genetics, nutrition, and management practices (81). This may partly explain why Low-input production systems showed lower lameness prevalence, despite some herds being housed in tie-stall systems during winter, which restrict animal movement. Lameness and reproductive disorders are major causes of culling (82, 83), so the combined differences observed in fertility and locomotion may partly contribute to the greater productive lifespan observed in Low-input production systems. Their lower prevalence in Low-input production systems likely contributes to greater longevity. Cleanliness scores were also improved in low-input systems, likely reflecting lower stocking density and greater outdoor access (20, 35). Nevertheless, hygiene levels were globally acceptable across all production systems.

Taken together, these clinical indicators suggest that overall animal health status was good across the three production systems. These results suggest that animal health, reproduction, and longevity indicators varied across systems, but these differences should be interpreted cautiously in light of potential confounding factors, including breed composition, country-specific management practices, and housing conditions. Consequently, the observed differences should be interpreted as system-associated patterns rather than direct causal effects of production system on animal health outcomes.

### 4.5 Implications for agroecological livestock systems

In the European dairy sector, low-input grazing systems can be considered operational models of agroecology because they reduce external inputs, maximize grassland resources, and maintain breed diversity, thereby aligning with the principles proposed by Dumont et al. (7). The present results suggest that such systems can maintain satisfactory levels of animal health while supporting production under commercial farming conditions. In particular, the combination of lower lameness prevalence, shorter calving intervals, and greater cow longevity indicates that Low-input production systems may contribute to improved animal robustness and potentially reduced replacement needs. These observations are consistent with previous studies reporting associations between greater pasture access, lower production intensity, and improved animal welfare and robustness (7). Potential biological explanations may include a reduced metabolic burden allowing a greater allocation of resources toward maintenance and immune functions rather than maximal milk production (84), as well as the beneficial effects of softer walking surfaces on hoof health.

Beyond animal health, these findings have broader implications for the sustainability of dairy farming. By relying more heavily on grazing and locally available forage resources, Low-input production systems may reduce dependence on imported feed and external inputs, thereby improving resource-use efficiency and contributing to the maintenance of permanent grasslands and associated ecosystem services. In addition, the greater diversity of breeds observed in these systems may enhance resilience to environmental and economic challenges. However, these potential advantages should be considered alongside important trade-offs. Milk production per cow was substantially lower in the Low-input production system than in the more intensive production systems. Consequently, the economic performance of low-input systems may depend on specific market conditions, product valorization strategies (e.g., PDO-labelled products), production costs, and public support mechanisms. Furthermore, the lower productivity observed at the animal level may require larger land areas or different management strategies to maintain overall farm profitability. These results suggest that Low-input production systems represent a credible pathway for reconciling animal health, welfare, and agroecological objectives.

## 5 Conclusion

This study explores animal health, metabolic, and productive indicators across a gradient of dairy production systems representative of commercial farming conditions in Europe. Overall, dairy cow health appeared satisfactory across the three production systems, with most clinical, milk, and blood biomarkers remaining within commonly reported physiological reference ranges. Several differences were observed between production systems. In particular, Low-input production systems were associated with lower lameness scores, shorter calving intervals, higher body condition scores, and greater culling age compared with some of the more intensive systems. Differences in metabolic indicators were also observed, although most values remained within physiological ranges and did not indicate clinically relevant metabolic disorders. However, these benefits must be interpreted within the limitations of the study’s observational design, which features partial confounding and imbalances between countries, breeds, and herd sizes. To generalize these promising findings beyond the studied sample, future research must rely on larger, more diverse, and country-adjusted farm populations to better disentangle system-level effects from regional contextual noise.

## Data Availability

The original contributions presented in the study are included in the article/Supplementary material, further inquiries can be directed to the corresponding author.
